# Supplementary figures and images for: Chaperone-tip adhesin complex is vital for synergistic activation of CFA/I fimbriae biogenesis
Source: PLoS Pathog. 2020 Oct 2;16(10):e1008848. doi: 10.1371/journal.ppat.1008848 (PMC7531860; doi:10.1371/journal.ppat.1008848)

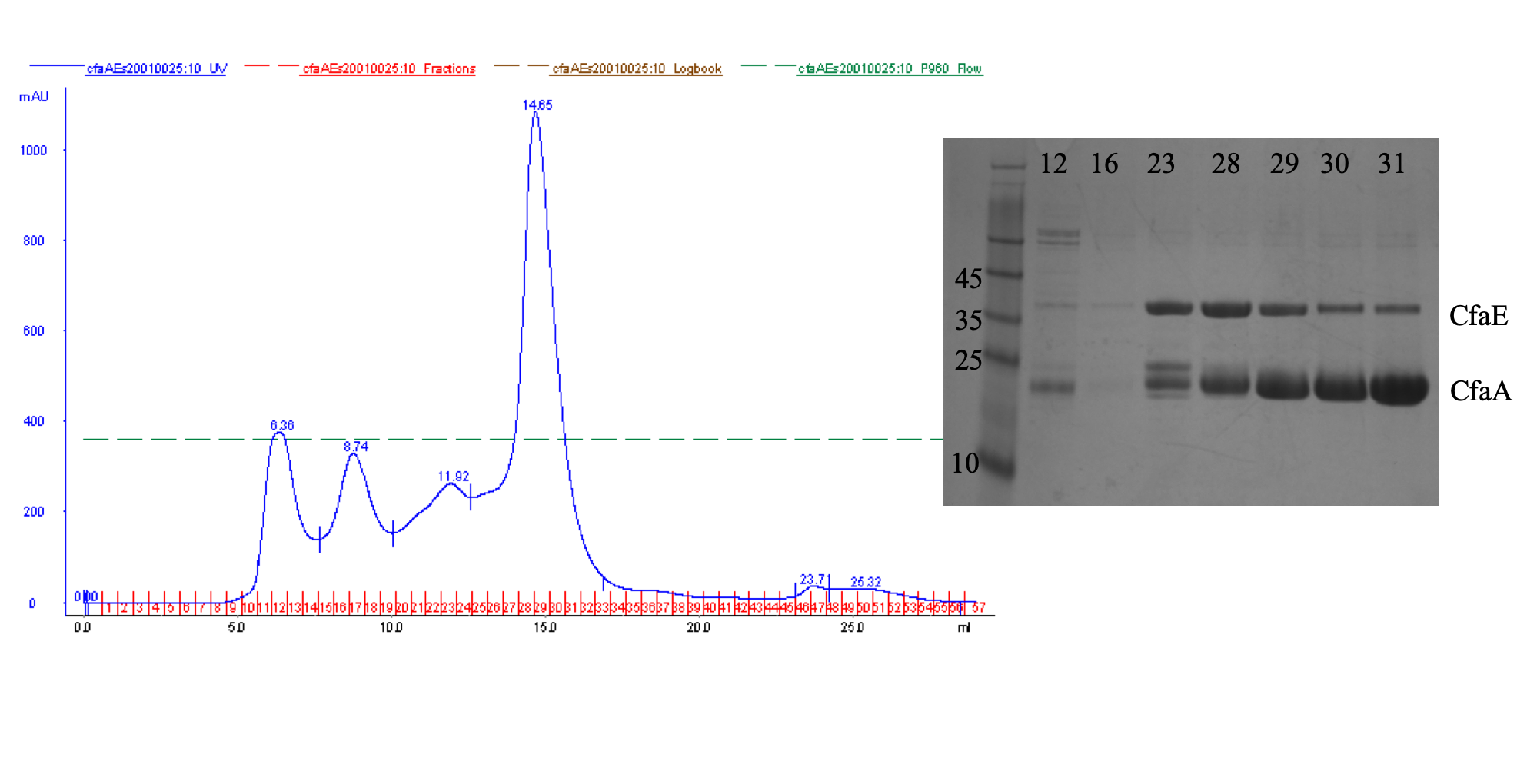

Supplement: S1 Fig — The fraction number is indicated at the top of SDS-PAGE. The results show that the native CfaA-CfaE is not stable and tends to dissociate in solution. (TIF) [file ppat.1008848.s001.tif]

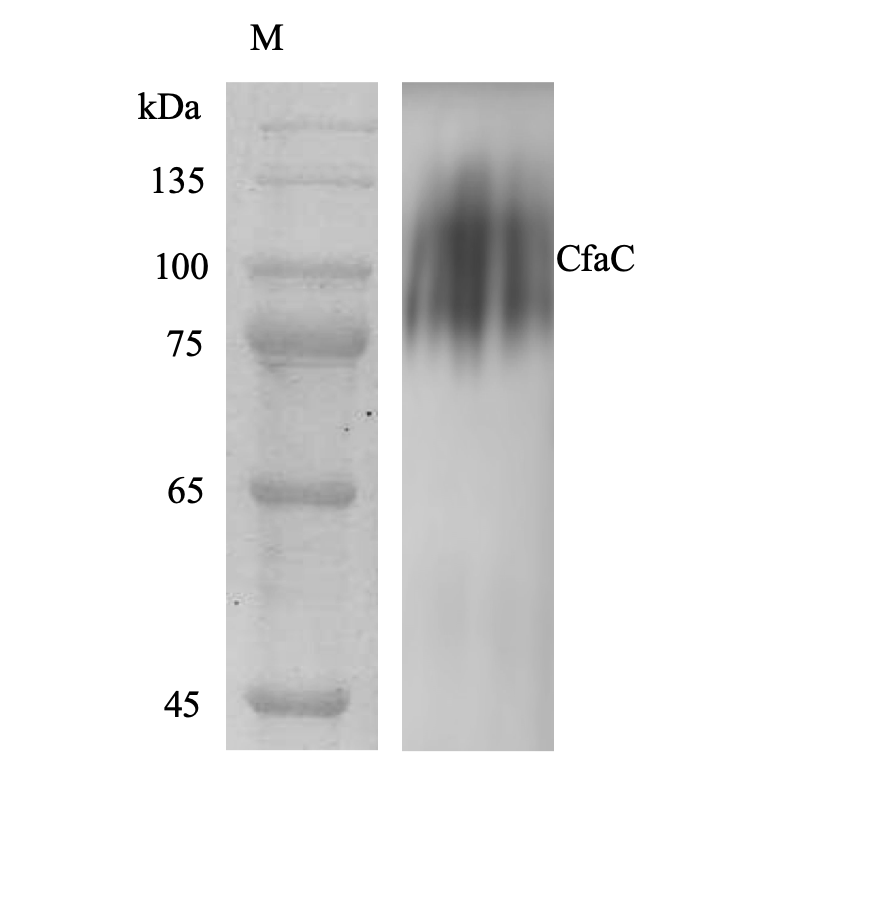

Supplement: S2 Fig — (TIF) [file ppat.1008848.s002.tif]

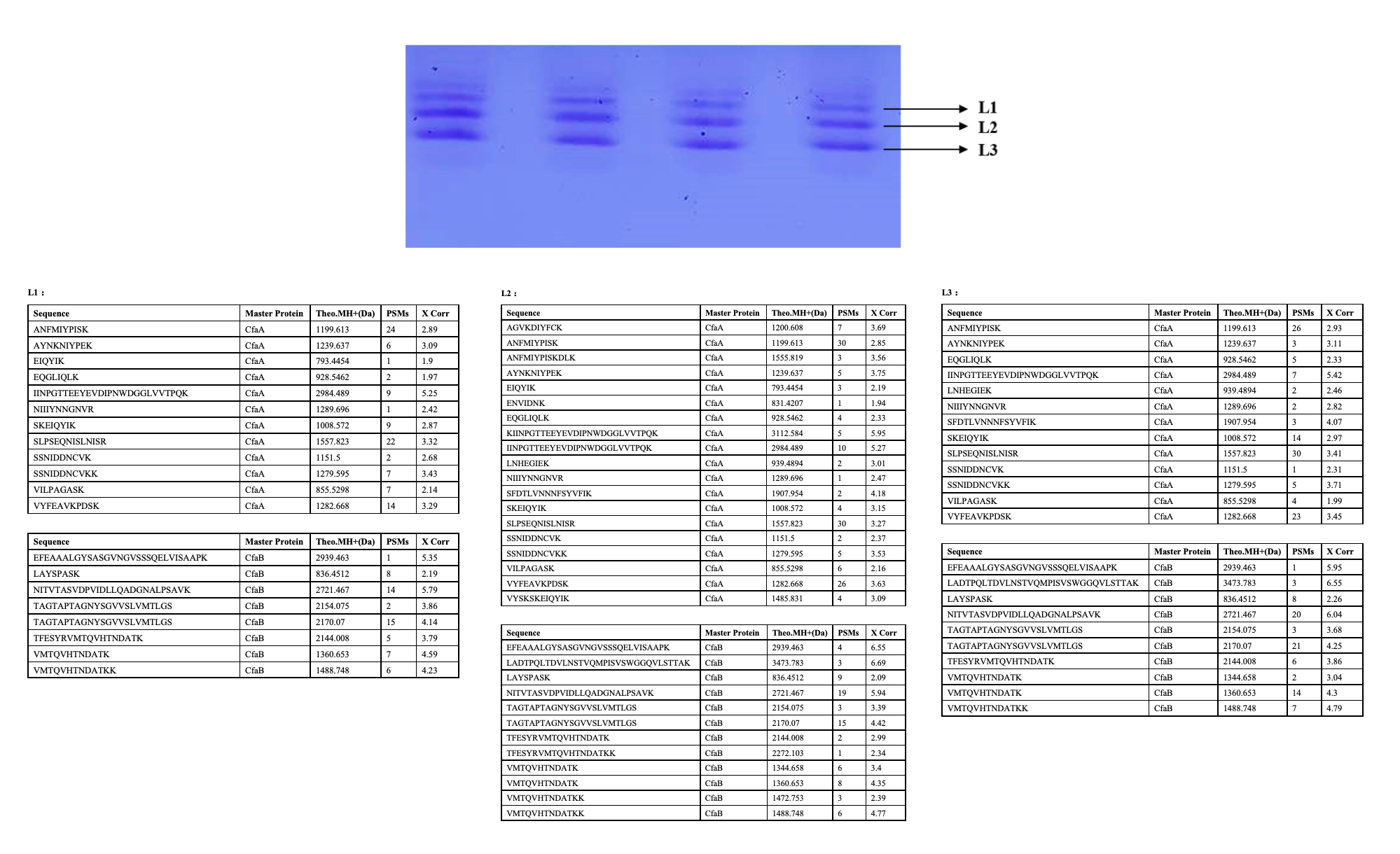

Supplement: S3 Fig — (TIF) [file ppat.1008848.s003.tif]
