# Supplementary material for: Chaperone-tip adhesin complex is vital for synergistic activation of CFA/I fimbriae biogenesis
Source: PLoS Pathog. 2020 Oct 2;16(10):e1008848. doi: 10.1371/journal.ppat.1008848 (PMC7531860; doi:10.1371/journal.ppat.1008848)
Supplement: S1 Table — (DOCX) [file ppat.1008848.s004.docx]

**S1 Table. Primers used to introduce mutations in pMAM2 in this work.**

| Mutants | Direction | Sequence |
| --- | --- | --- |
| pMAM2-CfaE/R253E | Forward | 5’-CAGCAGCTCTTTGGAGATAGAATTTCAGGATGATAATTCTAAA  TCTGATGG-3’ |
|  | Reverse | 5’-CCATCAGATTTAGAATTATCATCCTGAAATTCTATCTCCAAAG  AGCTGCTG-3’ |
| pMAM2-  CfaE/(R311-I320) A | Forward | 5’-GCTTCTCTGGAAACAAACTGGAATGCAGCTGCAGCTGCCGCC  GCGGCAGCAGCCAGTGTTCCGGTGTTGTGTTGGCC-3’ |
|  | Reverse | 5’-GGCCAACACAACACCGGAACACTGGCTGCTGCCGCGGCGGCA  GCTGCAGCTGCATTCCAGTTTGTTTCCAGAGAAGC-3’ |
| pMAM2-CfaE/S357Y | Forward | 5’- CTTTCACACCAAGTTATCAAACACTCTAGGACCCAGC-3’ |
|  | Reverse | 5’- GCTGGGTCCTAGAGTGTTTGATAACTTGGTGTGAAAG-3’ |
| pMAM2-CfaE/Q358A | Forward | 5’- CTTTCACACCAAGTAGTGCGACACTCTAGGACCCAGC-3’ |
|  | Reverse | 5’- GCTGGGTCCTAGAGTGTCGCACTACTTGGTGTGAAAG-3’ |
| pMAM2-CfaE/T359A | Forward | 5’-CACACCAAGTAGTCAAGCGCTCTAGGACCCAGC-3’ |
|  | Reverse | 5’- GCTGGGTCCTAGAGCGCTTGACTACTTGGTGTG-3’ |
| pMAM2-CfaE/L360A | Forward | 5’-CACACCAAGTAGTCAAACAGCCTAGGACCCAGCTTTC-3’ |
|  | Reverse | 5’- GAAAGCTGGGTCCTAGGCTGTTTGACTACTTGGTGTG-3’ |
| pMAM2-CfaE/Δ358-360) | Forward | 5’- CTTTCACACCAAGTAGTTAGGACCCAGCTTTCTTG-3’ |
|  | Reverse | 5’- CAAGAAAGCTGGGTCCTAACTACTTGGTGTGAAAG-3’ |
| pMAM2-ΔCfaE | Forward | 5’-GGATTTGCTGGCAAAGAATGATAAAGGATAAACGGACCCAG  CTTTCTTGTACAAAGTGGTGATGATCC-3’ |
|  | Reverse | 5’-GGATCATCACCACTTTGTACAAGAAAGCTGGGTCCGTTTATC  CTTTATCATTCTTTGCCAGCAAATCC-3’ |
