# Supplementary material for: Chaperone-tip adhesin complex is vital for synergistic activation of CFA/I fimbriae biogenesis
Source: PLoS Pathog. 2020 Oct 2;16(10):e1008848. doi: 10.1371/journal.ppat.1008848 (PMC7531860; doi:10.1371/journal.ppat.1008848)
Supplement: Attachment — Submitted filename: response-letter.docx [file ppat.1008848.s005.docx]

Dear Editor:

On behalf of all authors, I thank you for your letter and your detailed comments as well as those from the three referees. We followed these comments closely, checked our experimental results thoroughly and carefully, repeated some of the experiments, and revised the manuscript accordingly. Here we submit the revised manuscript along with a version with labeled modifications according to the instruction. The point-to-point responses to reviewers’ comments are given below. We hope that you and the reviewers will now find our revised manuscript satisfactory to be published in the journal PloS Pathogens. If you have any question, please don’t hesitate to let me know.

Sincerely yours.

Editor’s comments
*Reviewers #1 and #3 have raised multiple issues with the data presentation and interpretation and I recommend that you address their comments in full. Cartoons showing protein structures should be improved for visibility and clear annotations, as indicated by Reviewer #1. Points 4-6 raised by Reviewer #3 require that you undertake additional experiments to satisfy their concerns on the data quantification and reproducibility. In this regard, please refer to the data availability policy of PLoS Pathogens:*  [*https://journals.plos.org/plospathogens/s/data-availability*](https://journals.plos.org/plospathogens/s/data-availability)*. Raw data used to produce graphs and uncropped gel images should be made available as supplementary information or via DOI in public repositories.*

Thanks for your suggestions. We have improved the presentation in Figure 1B, 1C, 1D, 1E, Figure 2B, 2C, 2D, Figure 3A, 3B, Figure 4A, 4C, Figure 5B, Figure 6B according to reviewers’ comments. Experiments including (AN-PAGE and TEM) have been added or repeated to address the issues raised by the reviewers, the data have been uploaded according to PLoS Pathogens’ requirements.

*Please address Reviewers' #1 and #3 concerns on the quality of EM images, notably in Figure 1C, which at the present level of resolution do not support the claims on different pilus states-coiled versus extended. Please provide higher quality images and insets with higher magnification to clearly demonstrate the dimensions/diameters of different pili and show any differences between them. Pilus length measurements similar to those performed in Nishiyama et al 2008 would provide insight into pilus polymerization defects. Please indicate clearly the correspondence between samples in Figure 1B and 1C. It is unclear what the designation "uncatalyzed" refers to in your study.*

Following the recommendations from the editor and reviewers, we repeated the EM experiment, which led to the replacement of Figure 1B-E with images of better quality. From these EM micrographs, we can clearly distinguish the rods from the fibrils. We also did analysis of the images and provided measurements for the dimensions/diameters of the pili.

With respect to the designation of “uncatalyzed”, we defined the term “uncatalyzed” according to Nishiyama et al 2008, which means short pili self-assembled in the absence of chaperone or usher proteins. To better clarify this, we reorganized the Fig 1B-1C. In the revision, the images in Figure 1B illustrate the “uncatalyzed” CfaB in AN-PAGE gel and TEM.

*In the EM analysis of bacteria with mutations in CFA/I components, please indicate clearly which appendages correspond to CFA/I fimbriae and which ones are other pili/flagella. Please revise the model in Figure 6 following Reviewers #1 and #3 comments to summarize data described in the present study. Kinetic constants are not part of this research. Also, in the "off" pathway model, what is the reason that polymerization of CfaB fails to produce a coiled rod polymer in vitro? Are not all determinants of its molecular spring behaviour present in the major subunit (e.g. Spaudling et al eLife 2018)? Finally, may I request that you cite the appropriate original literature, notably reports obtained from related CU pili studies that set the paradigms for assembly mechanism, role of adhesin in pilus assembly initiation, usher dynamics, pilus rod spring-like properties etc.*

In our experiments evaluating impact of various mutations in CfaE to bacterial fimbriation, we used the *E. coli* strain BL21-AI (Thermo Fisher Scientific). This strain is one of the most extensively characterized *E. coli* strains. It is non-motile due to an IS element disrupting components for flagella biosynthesis(Jeong et al. 2009). This strain in the absence of CFA/I expression plasmid (pMAM2) or with component-removed CFA/I plasmid (such as the △CfaE as shown in Figure 5B) will not make any fimbriae. In the revision, a control figure representing untransformed BL21-AI strain is provided showing no surface pili and flagellar. Therefore, the observation of fimbriae in our EM micrographs is a result of pMAM2 or its mutants expressing and displaying CFA/I fimbriae on the surface of *E. coli*. Since the BL21 strain lacks flagellar biosynthesis, the large prominent surface structure is likely CFA/I pili bundled together.

We agree with the reviewer that this work does not address the kinetic aspect of the interaction between the CfaA-CfaE complex and CfaC. Thus, as suggested, we have revised Figure 6 (see figure below) by removing the kinetic parameters “K_fast_” and “K_slow_”. However, it is worth pointing out that both major subunit CfaB and minor subunit CfaE have very weak affinity for the wild-type chaperone CfaA at least in vitro. This property is unique to the CFA/I fimbriae and suggests an important role for the usher CfaC in the initiation of pilus assembly.

Previously, we showed that the CfaA-CfaB complex formed *in vivo* is not stable(Bao et al. 2016). The complex spontaneously dissociates and CfaB subunits are able to form trimer (CfaB)_3_ in solution, as detected by Blue-Native PAGE, which also happened even in the absence of CfaA. However, CfaA is able to delay the formation of the trimer. Unlike other periplasmic chaperones, CfaA only mildly accelerates the refolding of denatured CfaB. Mutations in CfaA that stabilized the CfaA-CfaB complex in vitro led to defects in fimbriation in vivo. Thus, it was proposed that the role of chaperone CfaA is to kinetically trap CfaB to form a metastable complex for efficient assembly by avoiding off-pathway self-assembly.

In this work, we want to establish an in vitro assay for the assembly of CFA/I fimbriae so that the function of CfaE and CfaC can be investigated. We used EM as a detection method for the pilus formation, because this method is capable of visualizing larger molecular assemblies. Clearly, the assembly of CfaB into a functional rod-like structure requires the functions of both CfaE and CfaC. In the absence of either component, the EM detects only short fibrils formed spontaneously, which is why the off-pathway assembly fails to produce functional coiled fimbriae. Conceivably, the spiral configuration for a functional CFA/I rod requires the CfaE adhesin to occupy the first tip site, the CfaC usher to guide the translocation of donor strand-exchanged CfaB subunits, and the intrinsic folding energy of CfaB to be utilized for its spiral extension on cell surface.

We agree with the editor that all determinants for a spring like behavior are in the CfaB subunit, which is reflected in our previous model of CFA/I pilus constructed based on crystal structures of CfaB, CfaBB, and CfaBBB that fit into an EM volume density(Li et al. 2009). That said, Mortezaei reported using AFM that CFA/I fimbriae are able to unwind under force and re-wind when the force is relaxed. However, different types of fimbriae require different forces for unwinding. CFA/I fimbriae require the least amount of force to unwind, followed by CS20 fimbriae and then P-fimbriae, which require the highest unwinding force(Mortezaei et al. 2015). Presumably, the difference in unwinding force lies in the interactions between subunits of neighboring layers.

In the revision, we have thoroughly reviewed our literature citations and added original works leading up to CU pilus assembly mechanism.

Figure 6 The model of chaperone-adhesin/usher controlled CFA/I fimbriae assembly.

Reviewers’ comments

**Part I - Summary**

*Reviewer #1: This manuscript is an important contribution to the field of bacterial adhesion and in the longer term, use of fimbriae in vaccine development. The authors provide new data, rigorously obtained, that advance our understanding of ETEC adhesion, which is essential for advancing efforts to prevent diarrheal diseases.

However, the authors discuss kinetics of assembly as if they are presenting kinetic data, which do not appear to be part of this research. The distinction between data-supported statements and speculation on their part needs to be better delineated*

We thank Reviewer 1 for her encouragement and appreciation of our work. We agree with the reviewer that the discussion on kinetics of assembly is speculative and have thus removed in the revised manuscript all the discussions relating to the kinetic aspect of the interaction of CfaA-CfaE complex with CfaC.

*Reviewer #2: The paper " Chaperone-tip Adhesin Complex Is Vital for Synergistic Activation of CFA/I fimbriae biogenesis" by Li-hui He and colleagues describes an in vitro assay to form CFA/I fimbria assembly and provide insight to the process that drives fimbria formation. The scope of this work is indeed very important for the fimbriae community, and I find the conclusions exciting and novel. Overall, the work is described clearly in the manuscript, and the results are strongly supported by data, and the authors' methods are sound and strong.*

We thank the reviewer for his/her positive comments on this manuscript.

*Reviewer #3: The manuscript by He et al. examines the assembly mechanism of CFA/I fimbriae expressed by enterotoxigenic E. coli. CFA/I fimbriae are major colonization factors and are assembled by the chaperone-usher (CU) pathway. The authors extend previous structure-function analyses of these fimbriae by mutating the CfaA chaperone to allow for purification of stable chaperone-subunit complexes with CfaB (major subunit protein) and CfaE (tip-localized adhesin subunit). Using this system, the authors establish an in vitro assay to monitor fimbrial polymerization and they generate a crystal structure of the CfaB-CfaE complex. Overall, while the authors present important new findings for the CFA/I fimbriae, the manuscript is not well organized or presented, the data seem preliminary and insufficient to support the conclusions, and the results need to be placed in better context of what is known from structural/functional analyses of other CU systems. Additional comments on the manuscript are listed below.*

We appreciate the reviewer’s comments on potential significance of the manuscript and his pointing out inadequacies in the way our data were presented. In the revision, we strive to overcome these inadequacies by reorganizing the manuscript and by including additional experiments, as suggested by the reviewer.

**Part II – Major Issues: Key Experiments Required for Acceptance**

Reviewer #1:

*While no key experiments are required for acceptance, it is essential that the authors clarify their statements on the kinetics of fimbria assembly - what data support their analysis, and what is speculation?*

We agree with the reviewer that this paper lacks the data to describe the kinetic aspects of the interaction of CfaA-CfaE with CfaC in the CFA/I fimbria assembly. Thus, as suggested, we have revised Figure 6 (see figure above) by removing the kinetic parameters “K_fast_” and “K_slow_” from it. We also revised our discussion accordingly.

By integrating our previous work (Bao et al., 2016) with extensive studies of other CU pili(Nishiyama et al. 2005; Thanassi et al. 1998; Phan et al. 2011a; Waksman and Hultgren 2009; Remaut et al. 2008; Sakellaris et al. 1996; Pakharukova et al. 2015; Berry et al. 2014; Zavialov et al. 2003; Båga et al. 1987; Remaut et al. 2006), we speculate that the tip-locating CfaE is a determinant for the coiled assembly of CfaB because it initiates the assembly by recognizing and binding to the CfaC usher platform. CfaC is another determinant. As shown in this and previous works, both major subunit CfaB and minor subunit CfaE have very weak affinity for the wild-type chaperone CfaA when they are co-expressed *in vivo*. The stability of CfaA/E complex presumably sets the path for assembly. It also implies that the interaction between CfaA-CfaE and CfaC should be significantly stronger than that between CfaA-CfaB and CfaC. This property is unique to the CFA/I fimbriae and suggest an important role for the usher CfaC in the initiation of pilus assembly and control mechanism of the assembly.

*Reviewer #2:*

*I find the experiments performed in this study strong!

Reviewer #3:*

*1. The manuscript text and figures need significant improvements for organization, presentation and clarity. Improvements are also needed for better citation of references and to correct errors throughout.*

As suggested, we carefully revised the text and re-made the figures with better quality. We have updated the references to make sure that original studies in the assembly mechanisms of CU pili and CFA/I fimbriae are properly cited.

*2. Many of the conclusions are not sufficiently supported by the data and are excessively speculative in nature, including the model presented in Fig. 6. The authors do not sufficiently incorporate or acknowledge the extensive prior structural and functional work done on CU pathways.*

We sincerely appreciate the valuable comments by the reviewer about inclusion of correct references of original works. In the revision, we have checked each reference carefully and added more to support our conclusions.

The added references are list below.

| Line number | Reference |
| --- | --- |
| L82 | Wolf MK. Occurrence, distribution, and associations of O and H serogroups, colonization factor antigens, and toxins of enterotoxigenic Escherichia coli. Clinical microbiology reviews. 1997;10(4):569-84. |
| L86 | Sakellaris H, Balding DP, Scott JR. Assembly proteins of CS1 pili of enterotoxigenic Escherichia coli. Molecular microbiology. 1996;21(3):529-41.  Froehlich BJ, Karakashian A, Meisen LR, Wakefield JC, Scott JR. CooC and CooD are required for assembly of CS1 pili. Molecular microbiology. 1994;12(3):387-401.  Sakellaris H, Scott JR. New tools in an old trade: CS1 pilus morphogenesis. Molecular microbiology. 1998;30(4):681-7. |
| L87 | Mu X-Q, Savarino SJ, Bullitt E. The three-dimensional structure of CFA/I adhesion pili: traveler’s diarrhea bacteria hang on by a spring. Journal of molecular biology. 2008;376(3):614-20.  Andersson M, Björnham O, Svantesson M, Badahdah A, Uhlin BE, Bullitt E. A structural basis for sustained bacterial adhesion: biomechanical properties of CFA/I pili. Journal of molecular biology. 2012;415(5):918-28. |
| L89 | Mu X-Q, Savarino SJ, Bullitt E. The three-dimensional structure of CFA/I adhesion pili: traveler’s diarrhea bacteria hang on by a spring. Journal of molecular biology. 2008;376(3):614-20.  Sauer MM, Jakob RP, Eras J, Baday S, Eriş D, Navarra G, et al. Catch-bond mechanism of the bacterial adhesin FimH. Nature communications. 2016;7(1):1-13.  Le Trong I, Aprikian P, Kidd BA, Forero-Shelton M, Tchesnokova V, Rajagopal P, et al. Structural basis for mechanical force regulation of the adhesin FimH via finger trap-like β sheet twisting. Cell. 2010;141(4):645-55. |
| L95 | Busch A, Waksman G. Chaperone–usher pathways: diversity and pilus assembly mechanism. Philosophical Transactions of the Royal Society B: Biological Sciences. 2012;367(1592):1112-22.  Nishiyama M, Horst R, Eidam O, Herrmann T, Ignatov O, Vetsch M, et al. Structural basis of chaperone–subunit complex recognition by the type 1 pilus assembly platform FimD. The EMBO journal. 2005;24(12):2075-86.  Waksman G, Hultgren SJ. Structural biology of the chaperone–usher pathway of pilus biogenesis. Nature Reviews Microbiology. 2009;7(11):765-74.  Zavialov AV, Berglund J, Pudney AF, Fooks LJ, Ibrahim TM, MacIntyre S, et al. Structure and biogenesis of the capsular F1 antigen from Yersinia pestis: preserved folding energy drives fiber formation. Cell. 2003;113(5):587-96.  Remaut H, Tang C, Henderson NS, Pinkner JS, Wang T, Hultgren SJ, et al. Fiber formation across the bacterial outer membrane by the chaperone/usher pathway. Cell. 2008;133(4):640-52.  Pakharukova N, Garnett JA, Tuittila M, Paavilainen S, Diallo M, Xu Y, et al. Structural insight into archaic and alternative chaperone-usher pathways reveals a novel mechanism of pilus biogenesis. PLoS pathogens. 2015;11(11).  Sauer FG, Fütterer K, Pinkner JS, Dodson KW, Hultgren SJ, Waksman G. Structural basis of chaperone function and pilus biogenesis. Science. 1999;285(5430):1058-61.  Choudhury D, Thompson A, Stojanoff V, Langermann S, Pinkner J, Hultgren SJ, et al. X-ray structure of the FimC-FimH chaperone-adhesin complex from uropathogenic Escherichia coli. Science. 1999;285(5430):1061-6.  Thanassi DG, Saulino ET, Lombardo M-J, Roth R, Heuser J, Hultgren SJ. The PapC usher forms an oligomeric channel: implications for pilus biogenesis across the outer membrane. Proceedings of the National Academy of Sciences. 1998;95(6):3146-51. |
| L107 | Sauer FG, Fütterer K, Pinkner JS, Dodson KW, Hultgren SJ, Waksman G. Structural basis of chaperone function and pilus biogenesis. Science. 1999;285(5430):1058-61.  Choudhury D, Thompson A, Stojanoff V, Langermann S, Pinkner J, Hultgren SJ, et al. X-ray structure of the FimC-FimH chaperone-adhesin complex from uropathogenic Escherichia coli. Science. 1999;285(5430):1061-6.  Sauer FG, Pinkner JS, Waksman G, Hultgren SJ. Chaperone priming of pilus subunits facilitates a topological transition that drives fiber formation. Cell. 2002;111(4):543-51. |
| L118-119 | Nishiyama M, Ishikawa T, Rechsteiner H, Glockshuber R. Reconstitution of pilus assembly reveals a bacterial outer membrane catalyst. Science. 2008;320(5874):376-9.  Schembri MA, Pallesen L, Connell H, Hasty DL, Klemm P. Linker insertion analysis of the FimH adhesin of type 1 fimbriae in an Escherichia coli fimH-null background. FEMS microbiology letters. 1996;137(2-3):257-63.  Anderson KL, Billington J, Pettigrew D, Cota E, Simpson P, Roversi P, et al. An atomic resolution model for assembly, architecture, and function of the Dr adhesins. Molecular cell. 2004;15(4):647-57.  Dodson KW, Pinkner JS, Rose T, Magnusson G, Hultgren SJ, Waksman G. Structural basis of the interaction of the pyelonephritic E. coli adhesin to its human kidney receptor. Cell. 2001;105(6):733-43.  Hultgren SJ, Lindberg F, Magnusson G, Kihlberg J, Tennent JM, Normark S. The PapG adhesin of uropathogenic Escherichia coli contains separate regions for receptor binding and for the incorporation into the pilus. Proceedings of the National Academy of Sciences. 1989;86(12):4357-61.  Li Y-F, Poole S, Rasulova F, McVeigh AL, Savarino SJ, Xia D. A receptor-binding site as revealed by the crystal structure of CfaE, the colonization factor antigen I fimbrial adhesin of enterotoxigenic Escherichia coli. Journal of Biological Chemistry. 2007;282(33):23970-80. |
| L107 | Delete [4] |
| L122 | Delete [8,9] |
| L143 | Li Y-F, Poole S, Rasulova F, McVeigh AL, Savarino SJ, Xia D. A receptor-binding site as revealed by the crystal structure of CfaE, the colonization factor antigen I fimbrial adhesin of enterotoxigenic Escherichia coli. Journal of Biological Chemistry. 2007;282(33):23970-80.  Liu Y, Esser L, Interlandi G, Kisiela DI, Tchesnokova V, Thomas WE, et al. Tight conformational coupling between the domains of the enterotoxigenic Escherichia coli fimbrial adhesin CfaE regulates binding state transition. Journal of Biological Chemistry. 2013; M112. 413534.  Ford B, Verger D, Dodson K, Volkan E, Kostakioti M, Elam J, et al. Structure of the PapD-PapGII pilin complex reveals an open and flexible P5 pocket. Journal of bacteriology. 2012; 06651-11. |
| L257 | Sauer FG, Fütterer K, Pinkner JS, Dodson KW, Hultgren SJ, Waksman G. Structural basis of chaperone function and pilus biogenesis. Science. 1999;285(5430):1058-61.  Choudhury D, Thompson A, Stojanoff V, Langermann S, Pinkner J, Hultgren SJ, et al. X-ray structure of the FimC-FimH chaperone-adhesin complex from uropathogenic Escherichia coli. Science. 1999;285(5430):1061-6.  Kuehn MJ, Ogg DJ, Kihlberg J, Slonim LN, Flemmer K, Bergfors T, et al. Structural basis of pilus subunit recognition by the PapD chaperone. Science. 1993;262(5137):1234-41. |
| L325 | Sakellaris H, Balding DP, Scott JR. Assembly proteins of CS1 pili of enterotoxigenic Escherichia coli. Molecular microbiology. 1996;21(3):529-41.  Bao R, Liu Y, Savarino SJ, Xia D. Off‐pathway assembly of fimbria subunits is prevented by chaperone CfaA of CFA/I fimbriae from enterotoxigenic E. coli. Molecular microbiology. 2016;102(6):975-91.  Hultgren SJ, Lindberg F, Magnusson G, Kihlberg J, Tennent JM, Normark S. The PapG adhesin of uropathogenic Escherichia coli contains separate regions for receptor binding and for the incorporation into the pilus. Proceedings of the National Academy of Sciences. 1989;86(12):4357-61.  Sakellaris H, Penumalli VR, Scott JR. The level of expression of the minor pilin subunit, CooD, determines the number of CS1 pili assembled on the cell surface of Escherichia coli. Journal of bacteriology. 1999;181(5):1694-7.  Leclerc J-M, Quevillon E-L, Houde Y, Paranjape K, Dozois CM, Daigle F. Regulation and production of Tcf, a cable-like fimbriae from Salmonella enterica serovar Typhi. Microbiology. 2016;162(5):777-88.  Sajjan US, Xie H, Lefebre MD, Valvano MA, Forstner JF. Identification and molecular analysis of cable pilus biosynthesis genes in Burkholderia cepacia. Microbiology. 2003;149(4):961-71.  Zavialov A, Berglund J, Knight SD. Overexpression, purification, crystallization and preliminary X-ray diffraction analysis of the F1 antigen Caf1M–Caf1 chaperone–subunit pre-assembly complex from Yersinia pestis. Acta Crystallographica Section D: Biological Crystallography. 2003;59(2):359-62.  Salih O, Remaut H, Waksman G, Orlova EV. Structural analysis of the Saf pilus by electron microscopy and image processing. Journal of molecular biology. 2008;379(1):174-87. |
| L518 | Phan G, Remaut H, Wang T, Allen WJ, Pirker KF, Lebedev A, et al. Crystal structure of the FimD usher bound to its cognate FimC–FimH substrate. Nature. 2011;474(7349):49. |
| L468 | Phan G, Remaut H, Wang T, Allen WJ, Pirker KF, Lebedev A, et al. Crystal structure of the FimD usher bound to its cognate FimC–FimH substrate. Nature. 2011;474(7349):49.  Geibel S, Procko E, Hultgren SJ, Baker D, Waksman G. Structural and energetic basis of folded-protein transport by the FimD usher. Nature. 2013;496(7444):243-6.  Ford B, Rêgo AT, Ragan TJ, Pinkner J, Dodson K, Driscoll PC, et al. Structural homology between the C-terminal domain of the PapC usher and its plug. Journal of bacteriology. 2010;192(7):1824-31. |
| L473 | Geibel S, Procko E, Hultgren SJ, Baker D, Waksman G. Structural and energetic basis of folded-protein transport by the FimD usher. Nature. 2013;496(7444):243-6. |

As also pointed out by other reviewers, our current data do not demonstrate the interactions between CfaA-CfaE and CfaC. Thus, we removed the kinetic constants from the summary Figure 6. Nevertheless, it is worth pointing out that our work does demonstrate that CfaA-CfaB alone is not sufficient to form functional, rod-like fimbriae (Figure 1). Wild-type CfaA-CfaE complex is short-lived, even more unstable compared to the CfaA-CfaB complex(Bao et al. 2016). In our previous study (Bao et al., 2016), it was shown that CfaB or CfaA-CfaB complex synthesized *in vivo* can form a head-to-tail assembly of CfaB trimer spontaneously in solution when analyzed by PAGE and crystallography, which was termed off-pathway assembly*.* These data allowed us to speculate that the CfaA-CfaE likely to be recognized by and the subsequent transfer of the CfaE to CfaC very quickly, whereas in the absence of CfaE bound to CfaC, the interaction between CfaC and CfaA/B is not likely to be productive.

This speculation is consistent with previous studies that reveal the general principle of the CU pilus assembly pathway (Nishiyama et al. 2005; Thanassi et al. 1998; Phan et al. 2011a; Waksman and Hultgren 2009; Remaut et al. 2008; Sakellaris et al. 1996; Pakharukova et al. 2015; Berry et al. 2014; Zavialov et al. 2003; Båga et al. 1987; Remaut et al. 2006), and from the works that indicated that tip adhesive subunits are critical for pilus assembly (Nishiyama et al. 2008; Schembri et al. 1996; Anderson et al. 2004; Dodson et al. 2001; Liu et al. 2013; Li et al. 2007; Hultgren et al. 1989b; Kuehn et al. 1991; Sajjan et al. 2003), leading us to incorporate our conclusions into the CFA/I fimbria pathway, which is summarized in a scheme shown in Figure 6.

3. *Better description is needed for what was previously known regarding the triple CfaA mutant chaperone used to stabilize the CfaA-B and CfaA-E complexes in this study. Has this specific mutant chaperone been demonstrated to be functional for assembly of CFA/I fimbriae in bacteria? More information is also needed regarding the CfaA-B crystal structure used in this study for comparison with the CfaA-E structure. Is the crystal structure of the CfaA-B complex from reference 10 (Bao et al, 2016)? That structure uses CfaA with a single mutation (T112I) rather than a triple mutation (correct?). If the chaperones are different, then this complicates the direct comparisons made in the current study.*

From our previous work on the structure and function of CfaA-CfaB complex(Bao et al. 2016), we know that suitable mutations have to be made on chaperone G1 strand in order to obtain the stable chaperone-subunit complex for our *in vitro* assay and crystallization. In this work, we show that the CfaA-CfaE complex formed *in vitro* is even more unstable, when purified, than the CfaA-CfaB complex, prohibiting structural characterization. To stabilize the CfaA-CfaE complex, we had two CfaA mutants to choose from, as shown in Figure 6A and Table 3 of Bao et al., 2016: the single mutation T112I, and the triple mutation T112I/L114I/V116I. The T112I mutant in CfaA resulted in a delayed fimbria assembly, while the triple mutation T112I/L114A/V116A is able to rescue the phenotype and displays a similar assembly behavior as the wild type (From the Figure 6C and Table 3 of Bao et al., 2016). Furthermore, the triple mutation T112I/L114I/V116I has a similar binding toward both CfaB and CfaE, as shown in Figure 5C of Bao et al., 2016. Since CfaAT112I/L114I/V116I could form stable complex with both subunits and yet generate relatively less adverse effect than CfaAT112I does, we decided to choose the CfaA with triple mutations for our *in vitro* studies in this work.

In the revised version, we added “However, for CfaA, the single mutation T112I resulted in delayed fimbriation, a phenotype that can be rescued by the triple mutation T112I/L114I/V116I. The latter displays a similar behavior in fimbriation as the wild-type CfaA.”

*4. More data is needed to support the in vitro fimbrial polymerization assay. The gel and EM images are of poor quality and the differences in fimbrial fibers stated in the text (length of fibers and extended vs. helical forms) are not apparent in the EM images. Also, more time points are needed to look between the 0 and 16 h reaction times and quantitative analysis is needed of the reaction to support the authors’ conclusions.*

Following the reviewer’s suggestion, we did more careful EM and AN-PAGE experiments and added more time points in AN-PAGE analysis (Figure 1).

*5. The denaturation/fluorescence data used to probe thermodynamic stability of the CfaA-B and CfaA-E complexes are difficult to interpret and not convincing (and the data should be moved to the main text rather than in the supplemental information). These data are insufficient to support the authors’ conclusions regarding relative stabilities of the complexes.*

We respectfully disagree with the reviewer on this point. Dissociation and unfolding of protein complexes can take place under various conditions including heat, pH, denaturants, etc. Although, there are many different ways to monitor unfolding process, intrinsic fluorescence change has often been used particularly for proteins that lack enzymatic activities. For example, in the article by (Prajapati et al. 1998), the steady-state tryptophan fluorescence has been extensively used to obtain information on the subunit dissociation and unfolding in solution. There are also articles describing experiments that evaluate changes in protein stability induced by guanidine chloride (GdnCl), as in (Chemes et al. 2013), (Akhtar et al. 2002), (Montagner et al. 2016), (Upadhyay et al. 2014) and (Rumfeldt et al. 2006). These studies showed that the variations in concentration of GdnCl has strong effects on dissociation, denaturation and interactions of protein complexes. Because the CfaA-CfaE and CfaA-CfaB complexes each has a set of aromatic residues at the interacting surface (W207 and F353 of CfaE and Y92 and Y120 of CfaA), our experiments using the GdnCl-induced Trp fluorescence change reliably show the changes in fluorescence as a function of GdnCl concentration, reflecting the stability of the protein complex. Combining the previous research and our results, we conclude that the CfaA and CfaE dissociates at lower concentration of GdnCl than the concentration at which they begin to unfold.

Following the reviewer’s recommendation, we moved the figure on the GdnCl concentration-dependent fluorescence change from Supplemental Material to Figure 2D. As shown in the figure, the curves are clearly biphasic for both ^mt^CfaA-CfaE and ^mt^CfaA-CfaB and the midpoint concentrations are used as indicators for dissociation and denaturation, respectively. Thus, the GdnCl concentration for CfaA-CfaB dissociation is 1.415 M, while that for CfaA-CfaE is 0.966M, indicating that the stability of the CfaA-CfaB binary complex is better than the CfaA-CfaE, which is also consistent with the structural analysis of CfaA-CfaB and CfaA-CfaE.

*6. The analyses of the CfaE mutants – agglutination, ELISA, EM – also need improvements, including sufficient biological replicates to allow for statistical analyses and better EM images.*

Results have been uploaded with better qualities.

**Part III – Minor Issues: Editorial and Data Presentation Modifications**

Reviewer #1: *More minor points include figures that are difficult to interpret - most drawings of the models have lines that are too thin to be easily followed, and the point being made in the text is sometimes not clearly shown in the figure. Specific examples are shown below, but the authors should also look at all the figures and try to make them easier to understand without significant zooming.*

Thanks for the suggestion. The Figures have been re-made, taking into account of your critique.

*The authors state “CFA/I fimbriae exhibit a spring-like property, also
71 known as catch-bond, that allows persistent attachment to the intestinal
72 epithelia even under the conditions of shear stress[4].”
Catch-bonds are not ‘spring-like’, so this sentence appears to be incorrect.*

Thanks for pointing out the mistake, we have corrected in the revision in the Line 86-90.

In the revision, this sentence has been changed to “The helical form gives CFA/I fimbriae a spring-like property, being able to unwind and re-wind(Mu et al. 2008; Andersson et al. 2012). In addition, the CfaE adhesin displays shear force-enhanced receptor binding, which is similar to the catch bond used by FimH in Type-1 pili (Mu et al. 2008; Sauer et al. 2016; Le Trong et al. 2010). These properties allow persistent attachment to the intestinal epithelia.

*The authors state “the chaperone captures an unfolded pilus subunit in the
79 periplasm via the donor-strand complementation (DSC) mechanism”
Is the subunit really unfolded before attaching to the chaperone? Is this known?*

Although it is clear that pilus subunits are translocated across the plasma membrane via the SecYEG translocon in an unfolded state (Pugsley 1993), it is not so clear whether periplasmic chaperones interact directly with the unfolded peptides as they enter the periplasm. We agree with the reviewer that direct experimental evidence has yet to be obtained to show the interaction between chaperone and unfolded peptide. That said, circumstantial data indicate the possibility that such interaction does take place. Many groups demonstrated the ability of periplasmic chaperones in catalyzing or assisting subunit refolding (Soto et al. 1998; Sauer et al. 1999; Zavialov et al. 2003; Hung et al. 1999; Di Yu et al. 2012; Kuehn et al. 1991; Jones et al. 1997; Choudhury et al. 1999; Barnhart et al. 2000; Jacob-Dubuisson et al. 1994; Vetsch et al. 2004). In one of our previous publications (Bao et al., 2016), we showed that CfaA is able to accelerate the folding of denatured CfaB by 10 folds. Hultgren et al. reported that processing of PapG prepilin by signal peptidase is enhanced by the presence of pilin chaperone(Hultgren et al. 1989a). This suggests that the chaperone interacts with the nascent precursor during translocation and may therefore catalyze the correct folding of pilus subunits.

In the revision, we replace “unfolded” with “incompletely folded” in this sentence.

*L150-152 is awkward. It would be easier to read if it said ‘only in the presence of both’ rather than simply ‘both’*

Thanks, we have corrected the sentence as suggested.

*Table 1 ‘Unit Cell’ Numbers in parentheses are not explained.*

The explanation for ‘Unit Cell’ Numbers have been added to the Table 1.

Fig 2C - I cannot determine what the authors are trying to show. Is this a perspective view rather than orthographic?
The very thin lines of this figure are difficult to see

Fig 2C is a superposition between ^mt^CfaA-CfaE and ^mt^CfaA-CfaB^ntd^, based on the C-terminal lobes of CfaA in both structures. It intends to show that the two lobes of CfaA in the ^mt^CfaA-CfaE structure open wider in order to accommodate the bound larger CfaE adhesin. To improve the presentation, we replace it with a new view angle and thicker lines.

*L271-272. Unclear where the C-term of the chaperone is. Having just a closeup in fig 3 does not help orient the reader*

Sorry for the unclear description. The C-terminal residues of the subunit interact with the cleft region of the chaperone forming the second site of interactions. We revised this sentence into “The cleft of the chaperone forms an anchorage site to accommodate the C-terminal residues of the subunit.” and add a “C-ter” label in Figure 3A.

*Fig 4A does not show about which axis the domains are rotated. The lines are too thin to be visible, and the yellow has very little contrast.*

To avoid misunderstanding, we regenerated Figure 4A and remove the angle indicators. In Table 2, the joint angle between the AD and PD is defined between the longest inertial vectors of each domain, according to previous study (Li et al. 2009).

*L337 please clarify ‘preserves its folding energy for assembly initiation’.
This appears to be the authors’ speculation, not a result.*

Thanks for pointing it out, we revised this sentence as “...but may also preserve its folding energy for ....”.

L 339-343 needs a figure to visualize this change.

In the revised version, we refer Figure 4A after the sentence “…wrapping around the joint and interacting with both adhesin and pilin domains” for illustration. In Figure 4A, the loop 301-321 is highlight with large size and in CfaA-CfaE, the dash style is to show the disordered state.

*Fig 4C is not understandable. First, where are the beta strands that are discussed in the text? How is the looped ‘wedged between’? Second, is ‘gray’ the tan or the blue? Whichever it is, what is the other color? The atoms shown in blue/red are confusing, because then it is difficult to tell whether they are from the magenta or tan subunit. The whole figure could be re-thought. Perhaps have them displayed separately and then together?*

In the revision, we replaced this figure with a new Figure 4C, adding a zoomed-out view. The close-up view of the domain interaction differences.

Figure 4C. Superposing the pilin domain of CfaE of the ^mt^CfaA-CfaE complex (magenta) with that of CfaB-CfaE complex (gray), the CfaE loop (residues 241-248) connecting β-strands B2 and C2 and shown in thicker lines involve in domain interactions.

*Table 3 - is mis-spelled*

Corrected.

*L404 ‘and’ should be ‘or’*

Corrected.

*L405 it is unclear what the authors mean by saying these residues ‘may play a regulatory role in interacting with the chaperone’. In addition, this statement belongs in the discussion, not results.*

As reviewer suggested, we removed this sentence from the Result section and revised the statement in Discussion section Line 444 “...its interaction with the CfaC usher. The most prominent difference between the structure of CfaA-CfaE and that of CfaA-CfaB is the adhesin domain of CfaE, which presumably is the first to bind the usher CfaC and to go across the usher pore, as seen in the structure of the initiation complex of Type 1 fimbriae(Phan et al. 2011b).”

*L418 should be Table 3*

Corrected.

*Fig 5 please state the scale bar size in the legend — it is not legible even at 3x zoom
Are the thicker filaments bundled pili, or do these bacteria have flagella?!*

We have added the statement of the scale bar size in the Figure legend.

In our pilus assembly experiments, the *E. coli* strain BL21-AI, a derivative strain from BL21 (DE3), was used. This strain is one of the most extensively characterized *E. coli* strains. According to a study “An investigation into the pathogenic properties of Escherichia coli strains BLR, BL21, DH5α and EQ1” (Chart et al. 2000), BL21 does not express any observable surface structures when grown in nutrient broth. This observation was confirmed by sequencing the whole genome of this strain(Jeong et al. 2009). Therefore, we speculate that the thicker filaments observed in EM are bundled CFA/I pili.

*Fig 1C and Fig 5 - The electron micrographs shown are acceptable with respect to showing the data they are said to show, but they are of poor quality and detract slightly from the manuscript.*

We re-perform the EM experiment, and in the revision, better quality micrographs are provided along with a new BL-21 (AI) control figure. And we have done EM experiment of Figure 5 again, the primary data was shown as below, and the manuscript micrographs have been replaced by better quality ones.

Figure 5B. The Negative stain EM images of CFA/I on the surface of bacteria containing CfaE mutations. The scale of each EM picture is indicated. The white arrows indicate CFA/I fimbriae. The scale bar is 500 nm, and the zoom is 10,000 x.

*L459-463 why would it be paradoxical that the less stable is also less stable as a heterodimer?*

Major subunit CfaB can be expressed in *E. coli* and purified(Bao et al. 2016). Unlike CfaB, CfaE, when expressed alone in a donor-strand uncomplemented state, is not stable(Poole et al. 2007). We knew from previous experiments that CfaA-CfaB complex are not stable, which led us to hypothesize that CfaA plays a role in trapping CfaB intermediates from going into a non-productive off-pathway assembly. Because CfaE subunits cannot survive by themselves, we thought the CfaA-CfaE complex should be more stable than it is for the CfaA-CfaB complex. It has been puzzling to us the functional implication of this observation. Accrodingtly we revised the se

*L463-467 I don’t see the logic that leads the authors to conclude that a subunit with more ‘strain’ would be less stable.*

Thanks for your carefully reading, we revised this sentence as “....CfaA traps CfaE in a higher energy state that makes dissociation easier ....”

*L 477 It is not supported that assembly beginning with CfaE is due to the reasons stated, that ‘correct initiation may need more input energy and that elongation is rapid after initiation. Indeed, the fact that there is no assembly without CfaE means that ‘correct initiation’ is always the case, and there are no data in the manuscript regarding the kinetics of assembly.*

We agree with the reviewer. In the revision, this sentence has been changed to “The fact that CfaA has a lower affinity toward CfaE, as compared to that for CfaB, seems to further suggest that (1) correct initiation of the fimbria assembly may need a suitable conformational configuration on the part of CfaE, which is obtained by associating with CfaA, and (2) once fimbria assembly is initiated, elongation ensues vary rapidly.”

*L 495-499 clarifies statements made earlier in the manuscript, so perhaps should move there (L 337; see above).*

*Without energy input in the bacterial periplasm, CU fimbria systems depend on the subunit folding energy for its assembly, which is accomplished by the function of chaperones acting as subunit-folding regulators to trap the subunits in high-energy folding intermediates and to prevent self-polymerization of subunits in the periplasm [10, 31-38].*

As the reviewer suggested, we have moved this part to the Result, after Line 300.

*L504 what does ‘insufficient’ mean in this context?*

Here we want to clarify that the in-vitro polymerization of CfaB subunits in CfaA-CfaB and CfaC mixture (Fig 1E) is not the functional CFA/I fimbriae and would not be able to translocate to the surface of bacteria. Interactions between CfaE and CfaC is required to move forward the assembly process. To clarify and to make this statement clearer, we have rephrased the sentence as “.... the interaction of the CfaA-CfaB complex with CfaC could not form functional CFA/I fimbriae.”

*L508-509 ‘fits the energy profile of the CfaA-CfaE complex’. What energy profile? Are there data to support this statement?*

According to the work by Geibel and colleagues (Geibel et al. 2013), which analyzed structures of both the initiation complex of Type 1 pili (FimD-FimC-FimH) and the elongation complex (FimD-FimC-FimF-FimG-FimH), they found that the adhesive domain of FimH inside the usher pore is more tightly held in the initiation complex than it is in the elongation complex. Energy calculation revealed a low-energy path for pilin subunit F and G going across the usher pore from the periplasm side to the extracellular side. Such a low-energy path does not exist for the adhesive domain of the FimH in the initiation complex, indicating, indicating differential energy requirement for the processes of fimbria initiation and extension. More importantly to us, the low-energy path requires the pilin subunits to rotate by approximately 120 degree as they pass through the usher pore presumably around the linker resides between subunits. This rotation is consistent with our previous hypothesis of the cis/trans transition of a proline in the linker(Li et al. 2009) and here CfaC would serve as a propyl isomerase. Thus, we speculate that for CFA/I assembly, such energy landscape across CfaC for pilin subunits may also exist.

To make this point clear, we revised the statement as “Unlike multi-subunit fimbriae for which the order of subunit incorporation into a growing pilus was shown to be pre-determined by specific interactions between different N-terminal extensions (Gd strands) and subunits (Rose et al. 2008; Puorger et al. 2008), the CFA/I system has only one type of Gd strand from CfaB. The initiation of fimbria assembly and subsequent pilus extension is conceivably determined by the characteristics of the CfaA-CfaE complex and its interaction with the CfaC usher. The most prominent difference between the structure of CfaA-CfaE and that of CfaA-CfaB is the adhesin domain of CfaE, which presumably is the first to bind the usher CfaC and to go across the usher pore, as seen in the structure of the initiation complex of Type 1 fimbriae(Phan et al. 2011b). ” And we also rephrased the sentence as “... In addition, structural studies on Type 1 fimbria initiation complex (FimD-FimC-FimH) and elongation complex (FimD-FimC-FimF-FimG-FimH) as well as PapC C-terminal domain shows that the domain binding inside the usher pore is more tightly held in the initiation complex than it is in the elongation complex [41, 59, 60]. Base on those observations, we hypothesize that the interaction of the CfaA-CfaE complex with CfaC leads to the displacement of the plug domain of CfaC and facilitate the binding of CfaE adhesin domain into the CfaC luman. This mechanism seems to be energetically demanding and fits the energetic profile of the Type 1 fimbria assembly(Geibel et al. 2013). ”

*Fig 6 - red star is not visible except after significant zoom.*

We changed the size of the red star to make it clearer.

*L783-784* *please state what structure (which proteins) was deposited as pdb 6K73. If it is CfaA/CfaE as stated in the abstract, then what is shown in Fig 6B? Please include the pdbs of all the structures shown in Fig 2B-2D*

We have added the PDB code used in the Figure legends. In the schematic diagram Figure 6B, CfaA-CfaE, CfaA-CfaB complex have been labeled.

*Fig 3 it is confusing to have the subunits be blue and magenta, and then the atoms be colored as blue and red, irrespective of which subunit they are from.*

We are sorry to make the reviewer confused, we have changed the color of atom to black in the manuscript.

*Fig S2 the x-axis is mislabeled - it should be GdnCl. Also, there should be vertical lines marking the positions of the 2 inflection points discussed in the text.*

The vertical lines have been added.

Figure 2D. Recording of changes in aromatic residue fluorescence at 320 nm as a function of concentration of GdnCl for ^mt^CfaA-CfaB^ntd^ (○) and ^mt^CfaA-CfaE (▼). The number 1 and 2 indicate the two inflection points in ^mt^CfaA-CfaB^ntd^ (solid line) and ^mt^CfaA-CfaE (dash line), respectively, and the thick lines are the fitted curves by Graphpad Prsim.

*Reviewer #2: I found the following mistake in the introduction on page 4, row 70-73: “CFA/I fimbriae exhibit a spring-like property, also known as catch-bond, that allows persistent attachment to the intestinal epithelia even under the conditions of shear stress [4].”

A catch-bond does not give rise to spring-like properties! The spring-like properties of CFA/I, that is the ability to unwind and rewind the shaft subunits, was shown in Prof. Bullitt’s work in 2012,* *A Structural Basis for Sustained Bacterial Adhesion: Biomechanical Properties of CFA/I Pili. Journal of Molecular Biology, 415(5), 918–928. A catch-bond modulaters the bond-receptor interaction since they can dynamically change the bond-strength when exposed to a tensile force. A catch-bond behaves, therefore, more like a finger-trap, the more load that is applied to the bond, the life-time of the bond-receptor complex will increase, up to the threshold break force.*

Thanks for your suggestion, we are sorry for this mistake, we have fixed this error in revised manuscript.

Reviewer #3: (No Response)

Reference

Akhtar, M. S., A. Ahmad, and V. Bhakuni. 2002. Guanidinium chloride-and urea-induced unfolding of the dimeric enzyme glucose oxidase. *Biochemistry* 41 (11):3819-3827.

Anderson, K. L., J. Billington, D. Pettigrew, E. Cota, P. Simpson, P. Roversi, H. A. Chen, P. Urvil, L. du Merle, and P. N. Barlow. 2004. An atomic resolution model for assembly, architecture, and function of the Dr adhesins. *Molecular cell* 15 (4):647-657.

Andersson, M., O. Björnham, M. Svantesson, A. Badahdah, B. E. Uhlin, and E. Bullitt. 2012. A structural basis for sustained bacterial adhesion: biomechanical properties of CFA/I pili. *Journal of molecular biology* 415 (5):918-928.

Båga, M., M. Norgren, and S. Normark. 1987. Biogenesis of E. coli Pap pili: PapH, a minor pilin subunit involved in cell anchoring and length modulation. *Cell* 49 (2):241-251.

Bao, R., Y. Liu, S. J. Savarino, and D. Xia. 2016. Off‐pathway assembly of fimbria subunits is prevented by chaperone CfaA of CFA/I fimbriae from enterotoxigenic E. coli. *Molecular microbiology* 102 (6):975-991.

Barnhart, M. M., J. S. Pinkner, G. E. Soto, F. G. Sauer, S. Langermann, G. Waksman, C. Frieden, and S. J. Hultgren. 2000. PapD-like chaperones provide the missing information for folding of pilin proteins. *Proceedings of the National Academy of Sciences* 97 (14):7709-7714.

Berry, A. A., Y. Yang, N. Pakharukova, J. A. Garnett, W.-c. Lee, E. Cota, J. Marchant, S. Roy, M. Tuittila, and B. Liu. 2014. Structural insight into host recognition by aggregative adherence fimbriae of enteroaggregative Escherichia coli. *PLoS pathogens* 10 (9).

Chart, H., H. Smith, R. La Ragione, and M. J. Woodward. 2000. An investigation into the pathogenic properties of Escherichia coli strains BLR, BL21, DH5α and EQ1. *Journal of applied microbiology* 89 (6):1048-1058.

Chemes, L. B., M. G. Noval, I. E. Sánchez, and G. de Prat-Gay. 2013. Folding of a cyclin box linking multitarget binding to marginal stability, oligomerization, and aggregation of the retinoblastoma tumor suppressor AB pocket domain. *Journal of Biological Chemistry* 288 (26):18923-18938.

Choudhury, D., A. Thompson, V. Stojanoff, S. Langermann, J. Pinkner, S. J. Hultgren, and S. D. Knight. 1999. X-ray structure of the FimC-FimH chaperone-adhesin complex from uropathogenic Escherichia coli. *Science* 285 (5430):1061-1066.

Di Yu, X., L. J. Fooks, E. Moslehi-Mohebi, V. M. Tischenko, G. Askarieh, S. D. Knight, S. MacIntyre, and A. V. Zavialov. 2012. Large is fast, small is tight: determinants of speed and affinity in subunit capture by a periplasmic chaperone. *Journal of molecular biology* 417 (4):294-308.

Dodson, K. W., J. S. Pinkner, T. Rose, G. Magnusson, S. J. Hultgren, and G. Waksman. 2001. Structural basis of the interaction of the pyelonephritic E. coli adhesin to its human kidney receptor. *Cell* 105 (6):733-743.

Geibel, S., E. Procko, S. J. Hultgren, D. Baker, and G. Waksman. 2013. Structural and energetic basis of folded-protein transport by the FimD usher. *Nature* 496 (7444):243-246.

Hultgren, S. J., F. Lindberg, G. Magnusson, J. Kihlberg, J. M. Tennent, and S. Normark. 1989a. The PapG adhesin of uropathogenic Escherichia coli contains separate regions for receptor binding and for the incorporation into the pilus. *Proceedings of the National Academy of Sciences* 86 (12):4357-4361.

———. 1989b. The PapG adhesin of uropathogenic Escherichia coli contains separate regions for receptor binding and for the incorporation into the pilus. *Proceedings of the National Academy of Sciences* 86 (12):4357-4361.

Hung, D. L., J. S. Pinkner, S. D. Knight, and S. J. Hultgren. 1999. Structural basis of chaperone self-capping in P pilus biogenesis. *Proceedings of the National Academy of Sciences* 96 (14):8178-8183.

Jacob-Dubuisson, F., R. Striker, and S. J. Hultgren. 1994. Chaperone-assisted self-assembly of pili independent of cellular energy. *Journal of Biological Chemistry* 269 (17):12447-12455.

Jeong, H., V. Barbe, C. H. Lee, D. Vallenet, D. S. Yu, S.-H. Choi, A. Couloux, S.-W. Lee, S. H. Yoon, and L. Cattolico. 2009. Genome sequences of Escherichia coli B strains REL606 and BL21 (DE3). *Journal of molecular biology* 394 (4):644-652.

Jones, C. H., P. N. Danese, J. S. Pinkner, T. J. Silhavy, and S. J. Hultgren. 1997. The chaperone‐assisted membrane release and folding pathway is sensed by two signal transduction systems. *The EMBO journal* 16 (21):6394-6406.

Kuehn, M. J., S. Normark, and S. J. Hultgren. 1991. Immunoglobulin-like PapD chaperone caps and uncaps interactive surfaces of nascently translocated pilus subunits. *Proceedings of the National Academy of Sciences* 88 (23):10586-10590.

Le Trong, I., P. Aprikian, B. A. Kidd, M. Forero-Shelton, V. Tchesnokova, P. Rajagopal, V. Rodriguez, G. Interlandi, R. Klevit, and V. Vogel. 2010. Structural basis for mechanical force regulation of the adhesin FimH via finger trap-like β sheet twisting. *Cell* 141 (4):645-655.

Li, Y.-F., S. Poole, K. Nishio, K. Jang, F. Rasulova, A. McVeigh, S. J. Savarino, D. Xia, and E. Bullitt. 2009. Structure of CFA/I fimbriae from enterotoxigenic Escherichia coli. *Proceedings of the National Academy of Sciences* 106 (26):10793-10798.

Li, Y.-F., S. Poole, F. Rasulova, A. L. McVeigh, S. J. Savarino, and D. Xia. 2007. A receptor-binding site as revealed by the crystal structure of CfaE, the colonization factor antigen I fimbrial adhesin of enterotoxigenic Escherichia coli. *Journal of Biological Chemistry* 282 (33):23970-23980.

Liu, Y., L. Esser, G. Interlandi, D. I. Kisiela, V. Tchesnokova, W. E. Thomas, E. Sokurenko, D. Xia, and S. J. Savarino. 2013. Tight conformational coupling between the domains of the enterotoxigenic Escherichia coli fimbrial adhesin CfaE regulates binding state transition. *Journal of Biological Chemistry*:jbc. M112. 413534.

Montagner, C., M. Nigen, O. Jacquin, N. Willet, M. Dumoulin, A. I. Karsisiotis, G. C. Roberts, C. Damblon, C. Redfield, and A. Matagne. 2016. The role of active site flexible loops in catalysis and of zinc in conformational stability of Bacillus cereus 569/H/9 β-lactamase. *Journal of Biological Chemistry* 291 (31):16124-16137.

Mortezaei, N., B. Singh, J. Zakrisson, E. Bullitt, and M. Andersson. 2015. Biomechanical and structural features of CS2 fimbriae of enterotoxigenic Escherichia coli. *Biophysical journal* 109 (1):49-56.

Mu, X.-Q., S. J. Savarino, and E. Bullitt. 2008. The three-dimensional structure of CFA/I adhesion pili: traveler’s diarrhea bacteria hang on by a spring. *Journal of molecular biology* 376 (3):614-620.

Nishiyama, M., R. Horst, O. Eidam, T. Herrmann, O. Ignatov, M. Vetsch, P. Bettendorff, I. Jelesarov, M. G. Grütter, and K. J. T. E. j. Wüthrich. 2005. Structural basis of chaperone–subunit complex recognition by the type 1 pilus assembly platform FimD. 24 (12):2075-2086.

Nishiyama, M., T. Ishikawa, H. Rechsteiner, and R. Glockshuber. 2008. Reconstitution of pilus assembly reveals a bacterial outer membrane catalyst. *Science* 320 (5874):376-379.

Pakharukova, N., J. A. Garnett, M. Tuittila, S. Paavilainen, M. Diallo, Y. Xu, S. J. Matthews, and A. V. Zavialov. 2015. Structural insight into archaic and alternative chaperone-usher pathways reveals a novel mechanism of pilus biogenesis. *PLoS pathogens* 11 (11).

Phan, G., H. Remaut, T. Wang, W. J. Allen, K. F. Pirker, A. Lebedev, N. S. Henderson, S. Geibel, E. Volkan, and J. Yan. 2011a. Crystal structure of the FimD usher bound to its cognate FimC–FimH substrate. *Nature* 474 (7349):49-53.

Phan, G., H. Remaut, T. Wang, W. J. Allen, K. F. Pirker, A. Lebedev, N. S. Henderson, S. Geibel, E. Volkan, and J. Yan. 2011b. Crystal structure of the FimD usher bound to its cognate FimC–FimH substrate. *Nature* 474 (7349):49.

Poole, S. T., A. L. McVeigh, R. P. Anantha, L. H. Lee, Y. M. Akay, E. A. Pontzer, D. A. Scott, E. Bullitt, and S. Savarino. 2007. Donor strand complementation governs intersubunit interaction of fimbriae of the alternate chaperone pathway. *Molecular microbiology* 63 (5):1372-1384.

Prajapati, S., V. Bhakuni, K. R. Babu, and S. K. Jain. 1998. Alkaline unfolding and salt‐induced folding of bovine liver catalase at high pH. *European journal of biochemistry* 255 (1):178-184.

Pugsley, A. P. 1993. The complete general secretory pathway in gram-negative bacteria. *Microbiology*

*Molecular Biology Reviews* 57 (1):50-108.

Puorger, C., O. Eidam, G. Capitani, D. Erilov, M. G. Grütter, and R. Glockshuber. 2008. Infinite kinetic stability against dissociation of supramolecular protein complexes through donor strand complementation. *Structure* 16 (4):631-642.

Remaut, H., R. J. Rose, T. J. Hannan, S. J. Hultgren, S. E. Radford, A. E. Ashcroft, and G. Waksman. 2006. Donor-strand exchange in chaperone-assisted pilus assembly proceeds through a concerted β strand displacement mechanism. *Molecular cell* 22 (6):831-842.

Remaut, H., C. Tang, N. S. Henderson, J. S. Pinkner, T. Wang, S. J. Hultgren, D. G. Thanassi, G. Waksman, and H. Li. 2008. Fiber formation across the bacterial outer membrane by the chaperone/usher pathway. *Cell* 133 (4):640-652.

Rose, R. J., D. Verger, T. Daviter, H. Remaut, E. Paci, G. Waksman, A. E. Ashcroft, and S. E. Radford. 2008. Unraveling the molecular basis of subunit specificity in P pilus assembly by mass spectrometry. *Proceedings of the National Academy of Sciences* 105 (35):12873-12878.

Rumfeldt, J. A., P. B. Stathopulos, A. Chakrabarrty, J. R. Lepock, and E. M. Meiering. 2006. Mechanism and thermodynamics of guanidinium chloride-induced denaturation of ALS-associated mutant Cu, Zn superoxide dismutases. *Journal of molecular biology* 355 (1):106-123.

Sajjan, U. S., H. Xie, M. D. Lefebre, M. A. Valvano, and J. F. J. M. Forstner. 2003. Identification and molecular analysis of cable pilus biosynthesis genes in Burkholderia cepacia. *Microbiology* 149 (4):961-971.

Sakellaris, H., D. P. Balding, and J. R. Scott. 1996. Assembly proteins of CS1 pili of enterotoxigenic Escherichia coli. *Molecular microbiology* 21 (3):529-541.

Sauer, F. G., K. Fütterer, J. S. Pinkner, K. W. Dodson, S. J. Hultgren, and G. Waksman. 1999. Structural basis of chaperone function and pilus biogenesis. *Science* 285 (5430):1058-1061.

Sauer, M. M., R. P. Jakob, J. Eras, S. Baday, D. Eriş, G. Navarra, S. Berneche, B. Ernst, T. Maier, and R. Glockshuber. 2016. Catch-bond mechanism of the bacterial adhesin FimH. *Nature communications* 7 (1):1-13.

Schembri, M. A., L. Pallesen, H. Connell, D. L. Hasty, and P. Klemm. 1996. Linker insertion analysis of the FimH adhesin of type 1 fimbriae in an Escherichia coli fimH-null background. *FEMS microbiology letters* 137 (2-3):257-263.

Soto, G. E., K. W. Dodson, D. Ogg, C. Liu, J. Heuser, S. Knight, J. Kihlberg, C. H. Jones, and S. J. Hultgren. 1998. Periplasmic chaperone recognition motif of subunits mediates quaternary interactions in the pilus. *The EMBO journal* 17 (21):6155-6167.

Thanassi, D. G., E. T. Saulino, and S. J. Hultgren. 1998. The chaperone/usher pathway: a major terminal branch of the general secretory pathway. *Current opinion in microbiology* 1 (2):223-231.

Upadhyay, A. K., A. Singh, K. Mukherjee, and A. K. Panda. 2014. Refolding and purification of recombinant L-asparaginase from inclusion bodies of E. coli into active tetrameric protein. *Frontiers in microbiology* 5:486.

Vetsch, M., C. Puorger, T. Spirig, U. Grauschopf, E. U. Weber-Ban, and R. Glockshuber. 2004. Pilus chaperones represent a new type of protein-folding catalyst. *Nature* 431 (7006):329-333.

Waksman, G., and S. J. Hultgren. 2009. Structural biology of the chaperone–usher pathway of pilus biogenesis. *Nature Reviews Microbiology* 7 (11):765-774.

Zavialov, A. V., J. Berglund, A. F. Pudney, L. J. Fooks, T. M. Ibrahim, S. MacIntyre, and S. D. Knight. 2003. Structure and biogenesis of the capsular F1 antigen from Yersinia pestis: preserved folding energy drives fiber formation. *Cell* 113 (5):587-596.
