# Supplementary material for: Chaperone-tip adhesin complex is vital for synergistic activation of CFA/I fimbriae biogenesis
Source: PLoS Pathog. 2020 Oct 2;16(10):e1008848. doi: 10.1371/journal.ppat.1008848 (PMC7531860; doi:10.1371/journal.ppat.1008848)
Supplement: Attachment — Submitted filename: response letter-revision 2.docx [file ppat.1008848.s006.docx]

Dear Editor:

On behalf of all authors, I thank you for your letter and your detailed comments as well as those referees. We followed these comments closely, and revised the manuscript accordingly. Here we submit the revised manuscript along with a version with labeled modifications according to the instruction. The point-to-point responses to reviewers’ comments are given below. We hope that you and the reviewers will now find our revised manuscript satisfactory to be published in the journal PloS Pathogens. If you have any question, please don’t hesitate to let me know.

Sincerely yours.

Editor’s comments

*In the case of Figure 1, which has been substantially modified, I suggest that you keep a similar mode of organisation as in the original submission, with the 4 figures showing the same type of in vitro assay data for four different protein samples grouped into one panel: SDS-PAGE data in Panel B, quantifications in Panel C, microcopy in D and measurements in E. Alternatively, you may choose to group the assay data and quantifications into a single panel and the EM images and quantifications in another. The figure legends should be modified accordingly and they should explain all the parts of the figures (this is not the case in the current version). For example, in the bottom panel of Fig 1B-1E, it is unclear why there is an inset in each panel with the quantification on a different scale. Are these different representations of the same data? Figure legends should provide more details on these graphs.*

Thanks for your suggestions. We have improved the Fig 1B-1E and modified the figure legends to explain all the parts of these Figures, and the modified Fig 1B-1E and the detailed figure legends are in the manuscript. As for the inset in the bottom panel of Fig 1B -1E, we would like to show as Figure 1 in “*Reconstitution of Pilus Assembly Reveals a Bacterial Outer Membrane Catalyst”,* which describes the length distribution histograms of the pili. And insert panels on a different scale show the frequency with pili length of 1-250nm. Combining the bottom panels and inset panels is more intuitive to describe the length of pili in different conditions.

*Please refer to the figures by "Fig 1A" instead of "Figure. 1A" etc. throughout the text.*

Thanks, we have corrected all the Figure X to Fig X.

*Please follow the same order of figures as that of the analyses presented in the main text. Thus, I suggest that the order of figures 4 and 5 be inverted, since you first refer to the data presented in Fig 5 in the main text, followed by those shown in Fig 4.*

Thanks. As you suggested, we have inverted the order of Fig 4 and Fig 5.

Minor editor's comments:

Line 63. We demonstrate

Corrected.
Line 64. Replace “play indispensable roles” with “is essential for “

Corrected.
Line 65. “The crystal structure of this complex reveals…”

Corrected.
Line 67. Remove “moreover” – Our findings suggest…

Corrected.
Line 69. …facilitates…

Corrected.
Line 70. …lumen. Collectively, our data demonstrate the critical role…

Corrected.
Line 117. “..tip-localized adhesins are required for initiation of assembly of various fimbriae…(24-29), including the Class 5 fimbriae (30)”

Corrected.
Line 131. “…mutant CfaA variant to stabilize…”

Corrected.
Line 137. Our work shows that CfaE is required for…

Corrected.
Line 157-160. When compared to native CfaA, the mutant variant containing the triple residue substitution T112I/L114I/V116I on the G1 strand (hitherto referred to as mtCfaA) formed significantly stabilized complexes with CfaE (Fig 1A), CfaB, and CfaBntd (the CfaB variant lacking the N-terminal donor strand).

Corrected.
Line 162. , 164 etc. (Fig S2) please correct all references to figures in this manner.

We have corrected the all Figure to Fig in the manuscript.
Line 168. (Fig 1B to Fig 1E top two panels) – referring to the figure in this way is confusing. Please show the data as a single figure file, with panels regrouped and labeled. The data from the current panels 1B to 1E should be regrouped.

We have modified Fig 1B-1E for more clearly understand the role of these proteins for the fimbriae assembly.
Line 178 …was able to prevent…

Corrected.
Table 1: Unit cell parameters

Corrected.
Line 213. … more open cleft…

Corrected.
Line 219. …shorter life-span compared to that of the CfaA-CfaB complex in solution.

Corrected.
Line 225. CfaA variant with triple residue substitution in the G1 strand (redundant as it has been introduced above)

Corrected.
Line 241. … allowed us to compare…

Corrected.
Line 260. … likely to engage in CfaA interaction (Fig 3A)

Corrected.
Also, please replace “C-terminal” by “C-terminus” in the figure 3A.

Corrected.
Line 269. Figure 5A data are described in the main text before Figure 4. The order of these figures should be inverted.

We have inverted the order of Fig 4 and Fig 5.

Line 381. Remove “the establishment” from the title and replace by "An in vitro system… etc"
Line 386. This mutant variant is able to…

Corrected.
Line 417. … suitable confirmation…

Corrected.
Line 419 …very rapidly…

Corrected.
Line 423. While mutations in these CfaE elements did not impair binding to CfeA, they affected assembly.

Corrected.
Line 425. …and not necessarily …

Corrected.
Line 452 ...do not require…

Corrected.
Line 455 …for the usher…

Corrected.
Line 456. … whether this motif is responsible….

Corrected.
Line 466 … show that the….

Corrected.
Line 467. … domain binds more tightly to the usher pore in the initiation complex compared to the elongation complex…

Corrected.
Line 468. Based on those….

Corrected.
Line 471. ....and facilitates binding of the CfaE adhesin domain to the CfaC lumen

Corrected.
line 623. concentrations ... were 0.25 etc.

Corrected.
line 647. …different mutations...

Corrected.
line 668. .. performed at 0.1M intervals

Corrected.
Line 686. modify the figure and the legend in the new version. Please explain the purpose of the two different scales and insets of the graphs on the bottom panels?

We have modified the figure and the legends in the new version. And firstly why we use two different scales in Fig 1B-1E is that CfaB self-assembly was very slow in the absence of mtCfaA-CfaE or CfaC, and no regular rod-like fimbriae were observed except for some short fragments. So, if the scale is the same as the other three, it may not be obvious or clear to be observed by TEM, therefore, we modulated the scale of the “Uncatatyzed” to 100 nm. And the reason why there are inset of the graghs in the bottom panels is that we would like to describe the pili length as Figure 1 in “*Reconstitution of Pilus Assembly Reveals a Bacterial Outer Membrane Catalyst*”, which used the length distribution histograms to show the frequency with pili length. And the insert panels are the frequency with pili length of 1-250nm. Therefore, combining the bottom panels and inset panels is more intuitive to describe the length distribution of pili in different conditions.

Line 691. ...ladders resulted from aggregation of...

Corrected.
Line 695... representative CfaB polymers...

Corrected.
Line 714. ... (dashed line) ... fitted curves generated by GraphPad...

Corrected.
Line 733. should be: "Superimposed cartoon models of the pilin domain CfaE from...

Corrected.
Line 737. The disordered loop 301-321 in mtCfaA-CfaE is shown as dashed line

Corrected.
Line 743. ... involved in domain interactions

Corrected.
Line 751. Remove the statement about the zoom.

Corrected.
Line 761. Two pathways are depicted.

Corrected.

Reviewer Comments (if any, and for reference):

Reviewer's Responses to Questions

**Part I - Summary**
Please use this section to discuss strengths/weaknesses of study, novelty/significance, general execution and scholarship.

Reviewer #1: The authors have successfully addressed all comments from the initial review, and the manuscript is acceptable for publication.

We thank the reviewer for the positive comments on this manuscript.

**Part II – Major Issues: Key Experiments Required for Acceptance**
Please use this section to detail the key new experiments or modifications of existing experiments that should be absolutely required to validate study conclusions.

Generally, there should be no more than 3 such required experiments or major modifications for a "Major Revision" recommendation. If more than 3 experiments are necessary to validate the study conclusions, then you are encouraged to recommend "Reject".
Reviewer #1: none.

**Part III – Minor Issues: Editorial and Data Presentation Modifications**
Please use this section for editorial suggestions as well as relatively minor modifications of existing data that would enhance clarity.

Reviewer #1:

L 63 demonstrate

Corrected.
L 64 plays

Corrected.

L 65 reveals

Corrected.
L 69 facilitates

Corrected.
L 140 functions

Corrected.
L 158 Figure

Corrected.
L 171 delete 'Fim'

Corrected.
L 178-179 I think you mean prevent self-assembly into off-pathway trimers -- it is 'self' assembing' (polymerizing) into short fimbria

Thanks. As you suggested, we have modified this sentence in the manuscript.
L346-347 it is not negative-stain EM, but EM of negatively stained fimbria

We have corrected in the manuscript.
L468 Based

Corrected.
L471 lumen

Corrected.

L 751 It is not clear what is meant by 'the zoom is 10,000x'. Was the image as sent to the journal zoomed 10kx? Since how it will be viewed or printed is not known, this is not useful information. If the authors would like to include something more than the scale bar, they can include the magnification at which the images were recorded.

Thanks. As you concerned, in addition the scale bar, we also want to indicate how many times it is enlarged. To avoid misunderstandings, we removed “the zoom is 10,000x” as editor’s suggestion and just kept the scale bar.
