# Supplementary material for: Chaperone-tip adhesin complex is vital for synergistic activation of CFA/I fimbriae biogenesis
Source: PLoS Pathog. 2020 Oct 2;16(10):e1008848. doi: 10.1371/journal.ppat.1008848 (PMC7531860; doi:10.1371/journal.ppat.1008848)
Supplement: Attachment — Submitted filename: revison response letter-4.docx [file ppat.1008848.s007.docx]

Dear Editor:

On behalf of all authors, I thank you for your letter and your detailed comments. We followed these comments closely, and the point-to-point responses are given below. We hope that you will now find our revised manuscript satisfactory to be published in the journal PloS Pathogens. If you have any question, please don’t hesitate to let me know.

Sincerely yours.

Editor’s comments

*We note that we have 6 Supporting Information files and 3 Supporting Information legends. Please ensure that all Supporting Information files are included correctly and that each one has a legend listed in the manuscript*

Thanks. We have confirmed all the Supporting Information files are included correctly and that each one has a legend listed in the manuscript, and just in case, we have re-uploaded a new Supporting Information file containing all seven Supporting Information legends.

*We note that the following authors have not yet confirmed their authorship on this manuscript: Tao Li, Changcheng Li, YiBo Zhu, and Yingjie Song.
We have re-sent their co-author confirmation email, containing an auto-confirmation link for them to click. If they have experienced any issues, please do ask them to contact us via email and we'd be happy to help. At this point the request is not urgent, all authors must confirm authorship before publication.*

Thanks. All of these four co-authors have confirmed the email.

*It is also important that any funding, and the role of Funders, is declared prior to review. Please ensure your Financial Disclosure statement is complete and accurate - amendments after review may not be permitted. For full instructions, please visit* [*https://journals.plos.org/plospathogens/s/submission-guidelines#loc-financial-disclosure-statement*](https://journals.plos.org/plospathogens/s/submission-guidelines#loc-financial-disclosure-statement)

Thanks for your advice. We have modified the Funding and the roles of Funders.
